# *In Situ* Mortality Experiments with Juvenile Sea Bass (*Dicentrarchus labrax*) in Relation to Impulsive Sound Levels Caused by Pile Driving of Windmill Foundations

**DOI:** 10.1371/journal.pone.0109280

**Published:** 2014-10-02

**Authors:** Elisabeth Debusschere, Bert De Coensel, Aline Bajek, Dick Botteldooren, Kris Hostens, Jan Vanaverbeke, Sofie Vandendriessche, Karl Van Ginderdeuren, Magda Vincx, Steven Degraer

**Affiliations:** 1 Bio-environmental research group, Institute for Agricultural and Fisheries Research, Oostende, Belgium; 2 Biology Department, Ghent University, Ghent, Belgium; 3 Department of Information Technology, Ghent University, Ghent, Belgium; 4 Ecloserie Marine de Gravelines, Gravelines, France; 5 Operational Directorate Natural Environment (OD Nature), Royal Belgian Institute of Natural Sciences (RBINS), Brussels, Belgium; Pacific Northwest National Laboratory, United States of America

## Abstract

Impact assessments of offshore wind farm installations and operations on the marine fauna are performed in many countries. Yet, only limited quantitative data on the physiological impact of impulsive sounds on (juvenile) fishes during pile driving of offshore wind farm foundations are available. Our current knowledge on fish injury and mortality due to pile driving is mainly based on laboratory experiments, in which high-intensity pile driving sounds are generated inside acoustic chambers. To validate these lab results, an *in situ* field experiment was carried out on board of a pile driving vessel. Juvenile European sea bass (*Dicentrarchus labrax*) of 68 and 115 days post hatching were exposed to pile-driving sounds as close as 45 m from the actual pile driving activity. Fish were exposed to strikes with a sound exposure level between 181 and 188 dB re 1 µPa^2^.s. The number of strikes ranged from 1739 to 3067, resulting in a cumulative sound exposure level between 215 and 222 dB re 1 µPa^2^.s. Control treatments consisted of fish not exposed to pile driving sounds. No differences in immediate mortality were found between exposed and control fish groups. Also no differences were noted in the delayed mortality up to 14 days after exposure between both groups. Our *in situ* experiments largely confirm the mortality results of the lab experiments found in other studies.

## Introduction

The increasing demand for renewable energy has led to innovative techniques and numerous ambitious projects. Offshore wind energy is particularly popular across the North Sea bordering countries. However, the construction of offshore wind farms - and the pile driving activities in particular - involve strong impulsive sounds, which are potentially harmful to marine fishes and more specifically to the early life stages of fishes, i.e. eggs, larvae and young juveniles [1], [2], [3], [4]. These early life stages are important as prey for pelagic fishes and for the recruitment to the adult fish populations, which stresses the need to understand how underwater sounds affect their fitness. Remarkably, many offshore wind farms are being installed or planned without extensive quantitative data on the physiological impact of strong impulsive pile driving sounds on fishes [2], [4].

In light of environmental impact control, it is necessary to establish sound level thresholds for pile driving at which fishes don't get injured. A first step in this process is to assess the sound level range causing immediate or delayed mortality [2]. Such assessments have been performed for Chinook salmon (*Oncorhynchus tschawytscha*), hybrid striped bass (white bass *Morone chrysops* x striped bass *Morone saxatilis*) and common sole (*Solea solea*), through lab experiments, using different methods to generate high-intensity ‘pile driving’ sounds in acoustically controlled chambers [5], [6], [7]. Juvenile Chinook salmon of 103 mm±8.75 (SD) exhibited mortal injuries, which include any mortality or injuries that can lead to mortality, at relatively high single-strike sound exposure levels (SEL_ss_) of 187 dB re 1 µPa^2^s, leading to a cumulative sound exposure level (SEL_cum_) of 220 dB re 1 µPa^2^s for 1920 strikes [6]. The hybrid striped bass of two different sizes; average 1.3 g and 17.2 g, exhibited mortal injuries at a SEL_ss_ of 180 dB re 1 µPa^2^.s for 960 strikes, resulting in a SEL_cum_ of 210 dB re 1 µPa^2^.s [7]. For common sole larvae that were exposed to a SEL_ss_ of 186 dB re 1 µPa^2^.s for 100 strikes, leading to a SEL_cum_ of 206 dB re 1 µPa^2^.s, no difference in mortality was found between control and exposed groups up to 7 days after exposure [5]. Since these laboratory experiments have not yet been verified in the field, there is a need to expose juvenile fishes to the sound exposure levels present in the immediate vicinity of the pile driving activity in order to examine direct or delayed mortality.

In the current study, a ‘worst-case scenario’ *in situ* field experiment was carried out to fill the gaps in the establishment of sound level thresholds for young fishes. Sound pressure was measured alongside a piling platform (45 m from the pile) and immediate and delayed mortality in young sea bass (*Dicentrarchus labrax*) were assessed.

## Material and Methods

### 1 Piling vessel and study location

**Figure 1:**
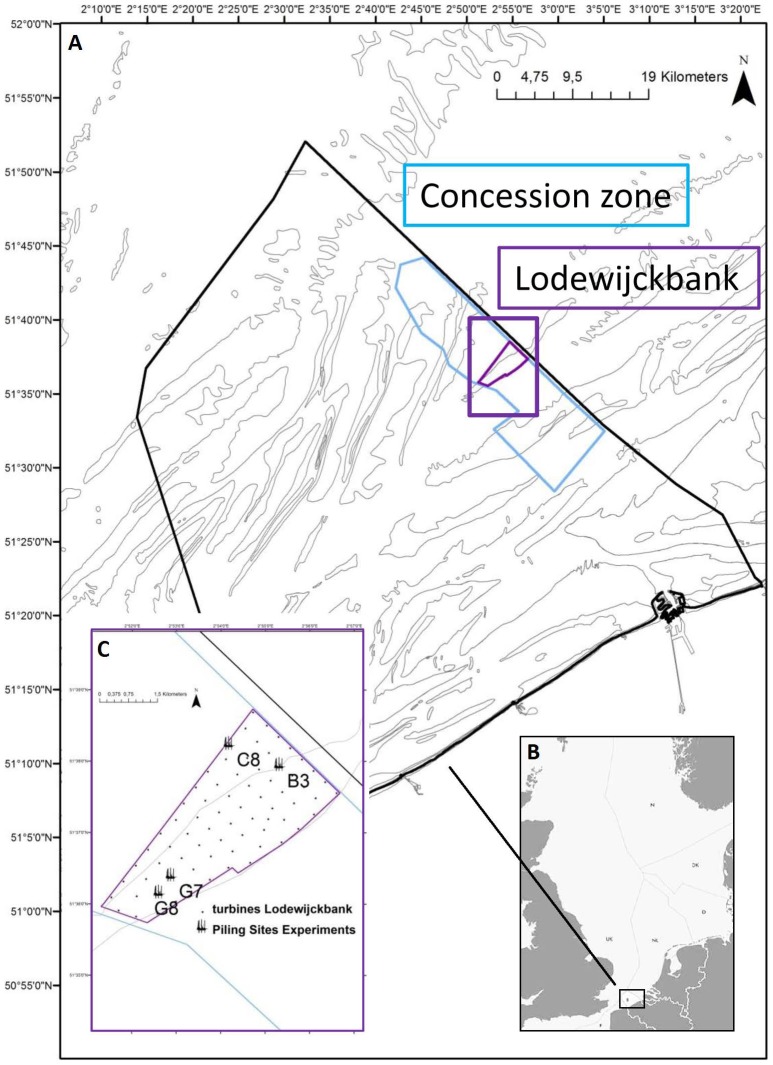
The offshore renewable energy zone in the Belgian part of the North Sea (a). North Sea exclusive economic zones (b). The Northwind concession on the Lodewijckbank includes 72 monopiles of ∼5 m diameter (c). The experiment was repeated at four monopiles (C8, B3, G7, G8).

To examine the impact of pile driving, this field study was performed on board of the jack-up piling vessel Neptune DP2 (GeoSea). Location was the Lodewijckbank, Belgian Part of the North Sea (Figure 1). This field experiment is part of the scientific research within the zone for renewable energy issued by the Belgian Ministry for the North Sea as described in the environmental permit NV Eldepasco [8].

Access to the deck side opposite to the pile driving activity was granted to perform the experiment, as close as 45 m from the monopile. Each monopile was designed for its specific position in the wind farm and varied in length, diameter and weight. Two monopiles were installed per trip using a hydraulic piling hammer (IHC Hydrohammer B.V.). Monopiles C8 (Lat N 51.637648, Long E 2.9003345, WGS84) and B3 (Lat N 51.629995, Long E 2.926765) were driven into the seabed during the first trip, monopiles G7 (Lat N 51.60667, Long E 2.881473) and G8 (Lat N 51.602782, Long E 2.877282) during the second trip (Table 1, Figure 1).

**Table 1:** Characteristics of the four monopiles and number of hammering strikes, energy and time of pile driving for each monopile.

Monopile number	C8	B3	G7	G8
Trip	1	1	2	2
Date	19/06/2013	19/06/2013	27/08/2013	28/08/2013
Start time (h)	1:47	18:38	22:39	11:15
Weight (tons)	471	357	346	346
Diameter (m)	5	5	5.2	5.2
Steel thickness (mm)	50	50	50	50
Length (m)	62	57	56	56
Depth in seafloor (m)	33	32	30	33
Total strikes	2282	2331	3249	2964
Maximum energy/strike (kJ)	1173	867	1069	1162
Total energy (kJ)	2333436	2276948	2333240	2526331
Total pile-driving time (h)	1:29	1:14	1:45	1:23
Net hammering time (h)	0:57	0:59	1:17	1:10
Time of day	1:47	18:38	11:15	22:39

### 2 Fish characteristics and preparation

European sea bass (*Dicentrarchus labrax*) is commercially exploited in the Southern North Sea and Mediterranean Sea, both through fisheries and aquaculture. Sea bass is a well-studied species, especially concerning larval growth, development and skeletal formation [9], [10], [11], [12], [13]. Swim bladder formation in sea bass larvae starts 6 **d**ays **p**ost **h**atching (dph) and is complete at 16 days. Sea bass is a physoclist round fish with an opening between the mouth and swim bladder only during the first days of the swim bladder formation [14]. Thereupon, buoyancy is controlled by gases retrieved from the blood, hence volume changes cannot be performed abruptly [15].

As sea bass eggs and larvae are available year round in the Ecloserie Marine de Gravelines (France), this largely facilitates the use of this species in experiments. This study was carried out in accordance with the Belgian Council for Laboratory Animal Science (BCLAS) guidelines. The experimental protocol was approved by the ethical committee of the Institute for Agricultural and Fisheries Research (ILVO) (Permit Number: 2012/178). The field study did not involve endangered or protected species.

Sea bass of 45 dph (hatched at the Ecloserie Marine de Gravelines) were incubated in cylindro-conic linear low-density polyethylene (LLDPE) containers of 9.5 L at ILVO. They were provided with aeration and a flow through of UV-sterilized sea water on a half-closed recirculation system. The fingerlings were kept in this aquaculture unit until the experiment could take place on board of the piling vessel. They were fed twice a day with Aglonorse (300-500 µm) or MariCo Start 1.5 mm (Coppens). The water temperature in the cultivation aquaria was 20.1°C±0.5, with a salinity of 30.2 ppt±0.1 and a pH of 8.1±0.1.

Fingerlings of 68 dph were used for the first *in situ* experiments on board of the piling vessel, while young sea bass of 115 dph from another batch were used during the second trip. Sea bass fingerlings were transported to the piling vessel during the first trip in 2 L containers and the second trip in 10 L buckets, each provided with oxygen tablets (JBL). After 3.5 hours, the seawater in the containers was renewed on board and a continuous air supply was provided. The sea bass were fed with Aglonorse (300–500 µm) or MariCo Start 1.5 mm (Coppens). Debris and dead fish were removed from the container and seawater was renewed on a daily basis to ensure good water conditions. Fish were checked twice a day and if they were in poor condition (showing illness, stress or decreased activity) they were not used in the experiments. Humane endpoints were incorporated if the fish would display behavioural abnormalities or haemorrhage. The fish would be humanely sacrificed by transferring them into an overdose anaesthetic ((2 ml of 5 g benzocaine dissolved in 25 ml aceton)/1 L seawater).

### 3 Acoustic equipment

Sound pressure was measured using a Brüel & Kjaer hydrophone (type 8104, voltage sensitivity 47.7 µV.Pa^−1^, charge sensitivity 0.391 pC.Pa^−1^, 10 m cable). The hydrophone was connected to the charge channel of a Brüel & Kjaer portable amplifier (Nexus type 2690-0S). The measurement chain was completed with a multi-channel portable recorder (Tascam DR-680). The signal was recorded in 1-channel WAVE format (.wav) on Compact Flash cards of 16 GB (SanDisk Ultra) with a sampling rate of 44 100 Hz at 24 bit.

### 4 Experimental setup and treatment

**Figure 2:**
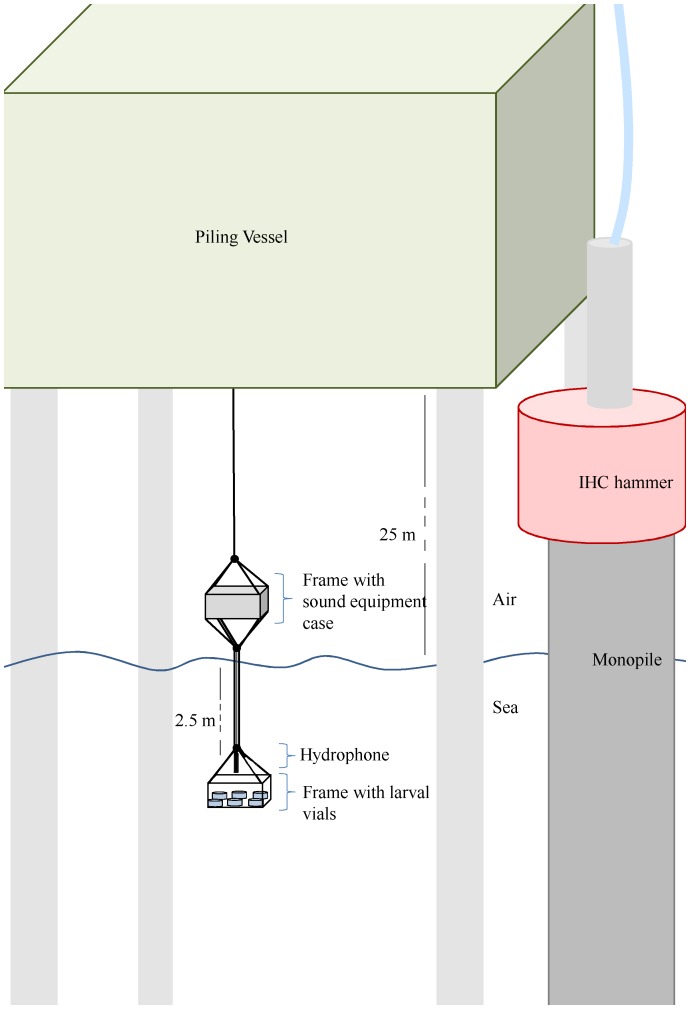
Experimental unit at the side of the piling vessel opposite to the monopile and hammering unit. The piling vessel is jacked-up 25 m above the sea surface on four steel piles. The upper frame above the sea surface holds the sound recording equipment. The lower frame holds the fish in vials. The hydrophone is attached between the steel cables of the lower frame.

The experimental unit existed of two parts: a stainless steel frame holding a case with the field recorder and amplifier; and a similar frame holding the six 500 ml vials with the fish (Figure 2). The vials were made of poly 4-methyl, 1- pentene (PMP) with an acoustic impedance of 1.84 Rayl, which is as close as possible to the impedance of seawater (1.56 Rayl) [16], [17]. The hydrophone was attached to the stainless steel cables holding the frames in such a way that it was hanging unobstructed 0.3 m above the vials. The whole experimental unit was lowered with a crane submerging the lowest frame to a depth of 2.5 m below the water surface.

Dead sea bass release toxins which negatively affect the survival of the others. To counteract this phenomenon, each treatment group (exposed and control) was subdivided into six vials per experiment. The number of fingerlings in the vials was based on their oxygen demand and on oxygen availability in the seawater in the vials, furthermore they were randomly assigned to a vial and group. During the first trip, each vial had a density of 20 individuals (68 dph; wet weight 42.8±15.8 mg; standard length 16.8±1.5 mm), whilst the density during the second trip was reduced to 2 individuals per vial (115 dph; 1613.3±472.5 mg; 47.9±4.5 mm). After transferring the fish into the vials, each vial was filled with care until a bubble of seawater would form on top of the vial. Then, the screw cap was screwed on the vial with a small overspill of seawater as a consequence. Each vial was checked for air bubbles before it went into the sea. After ∼1.5 hours (approximate duration of 1 monopile hammering event) the vials were inspected for immediate mortality, after which the fish were put together per two vials into a 2 L container with air supply. The same procedure was followed with the control group in the absence of pile driving and repeated for the following exposed and control groups, resulting in 4 experiments each. The total sample size was 528 individuals with 120 fish in each treatment group in the first two experiments and 12 fish per group in the third and fourth experiment.

After three days on board of the piling vessel, the fingerlings were transported back to the laboratory (see 2) and placed in cylindro-conical aquaria (9.5 L, first trip) or rectangular aquaria (30 L, second trip) per treatment and experiment. Delayed mortality was monitored twice a day during the following 14 days after exposure. 72 fish and four fish were followed for delayed mortality for each group respectively from the first and second trip, while the other 48 and eight of each group were stored in liquid nitrogen to analyse sub-lethal effects, and more precisely effects on stress hormone levels (Debusschere et al., in prep). At the end of the monitoring period, all fish were humanely euthanized.

### 5 Sound analyses

Metrics for the sound pressure *p* were calculated using Matlab R2012b (version 8.0).

The combination of single strike sound exposure level, cumulative sound exposure level and the total number of impulse events can be successfully correlated to the severity of fish injuries [6], [18]. In addition to these sound pressure metrics, the peak sound pressure level and the 1/3-octave band containing most energy were also calculated.

- The sound pressure level (SPL) is defined as the logarithmic measure of the instantaneous sound pressure within a given time interval. The unit is dB re 1 µPa.
- The peak sound pressure level (SPL_peak_) is defined as the level associated to the maximum absolute value of the instantaneous sound pressure within a given time interval. The unit is dB re 1 µPa.
- The single strike sound exposure level (SEL_ss_) is defined as the level associated to the integral of the squared sound pressure over the duration of a single impulse event. The unit is dB re 1 µPa^2^.s. Impulse events were detected on the basis of the SPL time series. The detection threshold was set to 170 dB, and single events were selected using a temporal window around the times that the threshold is exceeded, 0.20 s before the threshold and 0.50 s after. The minimum time between two events was set to 0.50 s. These optimal values were set after visual inspection of the recordings.
- The cumulative sound exposure level (SEL_cum_) is defined as the decibel sum of the sound exposure level over a number of individual impulse events. The time between the individual impulse events is excluded using this procedure.
- Given the average SEL_ss_ and the number of impulse events, the cumulative levels can be more practically (p) calculated [5], [6] as:

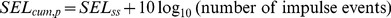
(1)


### 6 Statistical analyses

The Plymouth Routines In Multivariate Ecological Research (PRIMER) programme, version 6.1.12 with PERMANOVA add-on software, was applied for statistical analyses ([19]). A significance level of p <0.05 was used in all tests. The univariate permutational ANOVA's (PERMANOVA) were carried out with a 2 - factor design including treatment (T) and age (A) to analyse immediate mortality, while the delayed mortality over 14 days was analysed with a 3 - factor design, including treatment (T), age (A), and days after exposure (D) analysed in this order. The data were not transformed and Euclidean distance similarity matrices were applied. Due to the low replicate number the Monte Carlo P-value was preferred over the permutation P-value [20]. A simple linear regression analysed the potential linear relationship between total pile driving energy (Table 1) and SEL_cum_ (Table 2) using R version 2.15.1. Non-parametric Spearman rank tests were carried out to examine a correlation between the monopile characteristics (weight, length, penetration depth) and the pile driving characteristics (total energy, total strikes and maximum energy per strike). Furthermore, a non-parametric Spearman rank test was performed with penetration depth at each strike and energy per strike.

**Table 2:** Sound pressure metrics measured at 45 m during pile-driving of the four monopiles and for the control groups of each experiment.

Monopiles	C8	B3	G7	G8
Trip number	1	1	2	2
**Sound metrics to which the fish were exposed to**				
total strikes exposed to*	1739	2312	3067	2959
time between peaks (s)	1.49	1.49	1.39	1.41
SEL_ss_ mean (dB re 1 µPa^2^.s)	183	188	181	183
SEL_ss_ max (dB re 1 µPa^2^.s)	185	191	185	187
SEL_ss_ min (dB re 1 µPa^2^.s)	160	159	173	157
SPL_peak_ (dB re 1 µPa)	210	210	211	211
SEL_cum_ (dB re 1 µPa^2^.s)	215	222	217	218
SEL_cum,p_ (dB re 1 µPa^2^s)	215	222	216	218
1/3 octave band with most energy (Hz)	200	160	125	200
**Control replicate**	**1**	**2**	**3**	**4**
SPL (dB re 1 µPa)	138	128	145	136
1/3 octave band with most energy (Hz)	31.5	25	40	100

* Different from total number of strikes in table 1 as net time of exposure is less than total hammering time.

## Results

### 1 Sound parameters

No correlation was found between the monopile characteristics (weight, length, penetration depth, Table 1) and the pile driving characteristics (total energy, total strikes, and maximum energy per strike). Also, no linear relationship was found between the cumulative sound exposure level calculated for the total number of strikes (SEL_cum, p_) (Table 2) and the total energy necessary for one monopile (R^2^ = 0.14). On the other hand, the energy needed per strike was positively correlated with the penetration depth, which means that the type of sediment layers that have to be penetrated forms a key factor in terms of energy requirements (Spearman's rank correlation coefficient 0.94 (C8); 0.90 (B3); 0.94 (G7); 0.92 (G8)).

**Figure 3:**
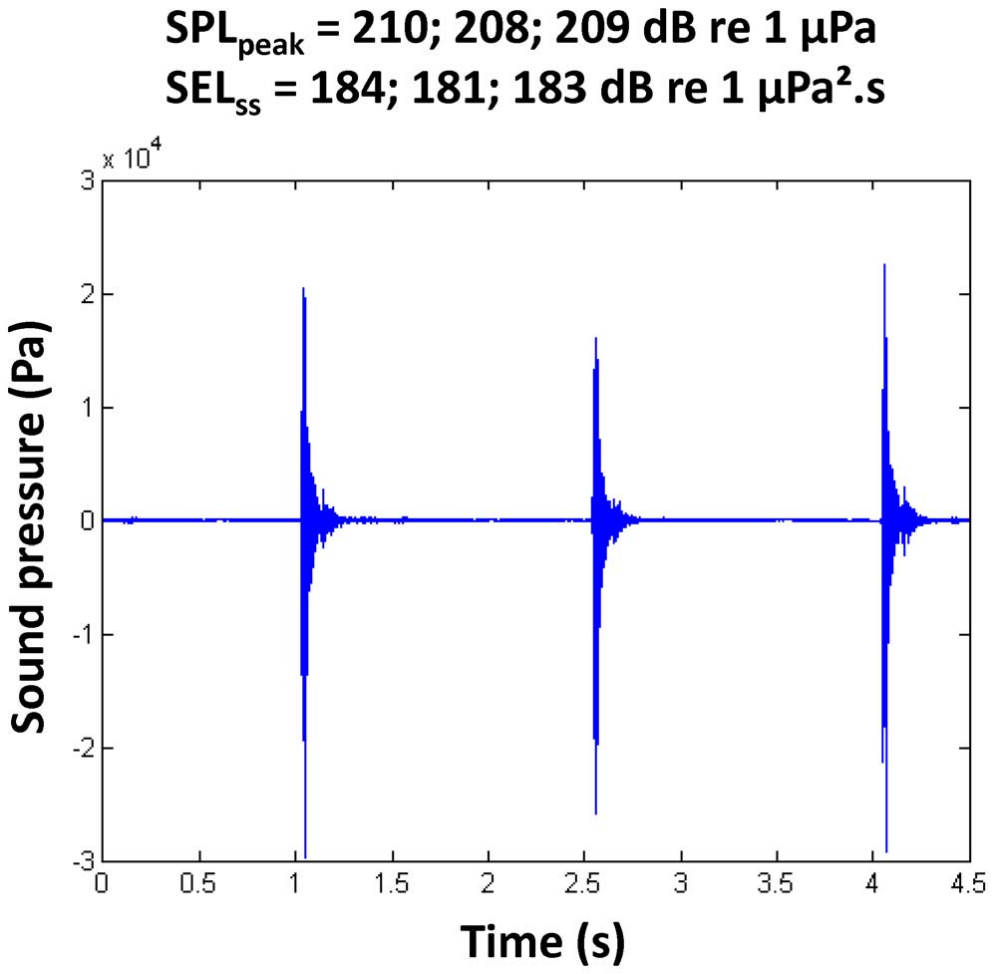
Detail of three consecutive piling strike signals. Detail of three consecutive piling strike signals recorded in the field for underwater sound pressure during experiment 3 (G7) (strike number 3005–3007 at 4892–4896.5 s).

**Figure 4:**
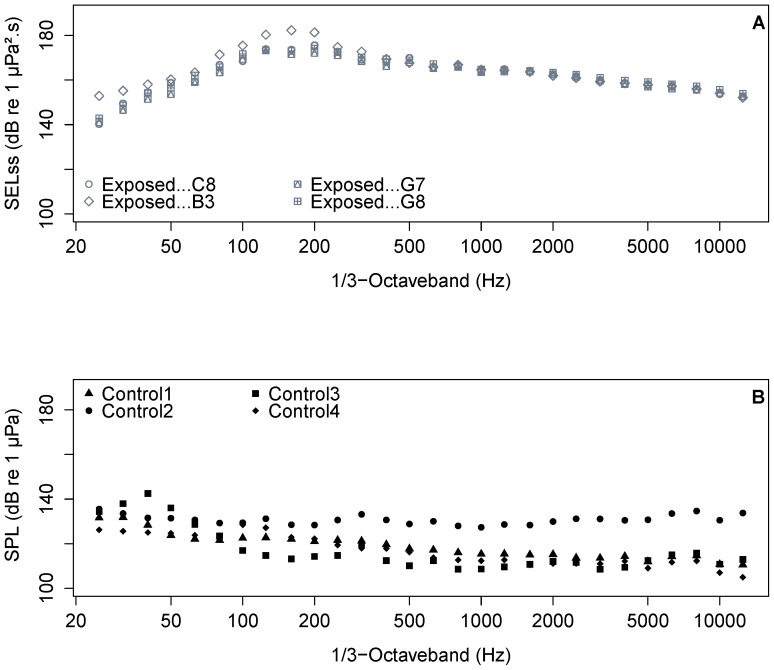
Measured frequency spectra in the presence and absence of pile driving. Mean SEL_ss_ (A) of the total recorded piling strikes versus 1/3 octave bands and SPL (B) of the control groups versus 1/3 octave bands. C8, B3, G7, G8 are the groups exposed to pile-driving; Control 1–4 are the control groups.

The pile driving sound levels that were measured during the four experiments at 2.5 m below the water surface reached on average SEL_ss_ = 181–188 dB re 1 µPa^2^s, rose to SPL_peak_ = 210–211 dB re 1 µPa^2^, and led to SEL_cum_ = 215–222 dB re 1 µPa^2^.s, with 1739 up to 3067 strikes per monopile (Table 2). The SPL_peak_ was constant across the four monopiles (see example in Figure 3), while B3 (experiment 2) had higher SEL_ss_ and SEL_cum_ values compared to the other three monopiles. The dominant energy during exposure (SEL_ss_) was present at 125–200 Hz, although no steep decline was recorded towards the higher frequencies (Figure 4a). For the experiments with control groups that were carried out in the absence of pile-driving activities on the piling vessel, only sound pressure level could be measured. The SPL varied between 127 and 145 dB re 1 µPa (Figure 4b).

### 2 Sea bass survival

**Figure 5:**
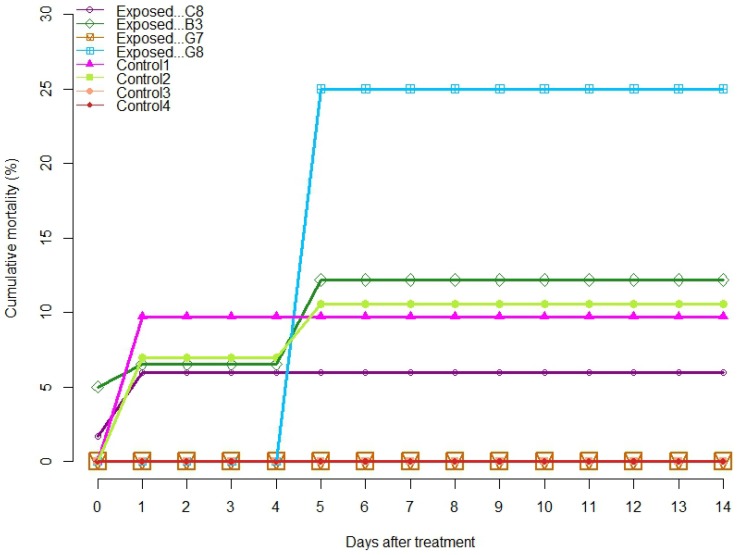
Cumulative mortality during 14 days after the experiment. Trip 1 (C8, B3, Control 1–2) and trip 2 (G7, G8, Control 3–4). All fish were transported back to the laboratory on day three.

During the first trip with the 68 dph fish (120 fish per treatment group), no immediate mortality was observed for the control groups, whereas a mortality of 1.7 and 5% was found for the exposure experiments 1 and 2, respectively (Figure 5). The dead fish were only seen in one of the six vials in experiment 1 and in two vials in experiment 2. During the second trip (experiments 3 and 4) with the 115 dph fish (12 fish per treatment group), no mortality was observed immediately after the experiments. The difference in immediate mortality (Monte Carlo p-value = 0.11) between control and exposed groups was not statistically significant. Transportation back to the lab did not cause direct mortality. At the end of the 14-day monitoring period of trip one, 9% of the exposed fish and 10.1% of the control fish died. As only four fish per group of the second trip were followed for delayed mortality, a dead fish caused a mortality increase of 25%. One fish of the exposed group died on day four and one fish of the control group on day 13, the latter jumped out of the aquarium and was not included in the statistical analysis. Delayed mortality did not differ significantly between the control and exposed groups of both trips (Monte Carlo p-value = 0.39).

## Discussion

### 1 Field exposure

As far as we know, this work on the effects of sound on fish presents the first *in situ* field study carried out as close as 45 m from offshore pile driving of monopiles. As such, our study is very valuable to validate the mortality results of laboratory experiments presented in other studies [5], [6], [15], [18]. Although, the underwater sound levels during the experiments with the control groups were above the background levels in the North Sea [21], due to sound radiation through the four jack-up piles of the piling vessel and the presence of the working vessels, these sound levels were unlikely to cause immediate or delayed mortality [4], [22], [23].

Both ages of sea bass that were used in our experiments are considered new fingerlings, but juveniles of 68 dph seem to be more sensitive in general (higher mortality) compared to juveniles of 115 dph. Still, both age groups largely survived the exposure to the high sound pressure levels that were exhibited over a complete piling session.

Exposure in the field to such sound levels did not cause significantly increased mortality during the first 14 days after exposure of sea bass *Dicentrarchus labrax* of 68 and 115 dph. This result strengthens the trend seen in the recent lab studies with the survival of common sole larvae (*Solea solea*) [5], juvenile Chinook Salmon (*Oncorhynchus tshawytscha*) [6], lake sturgeon (*Acipensen fulvescens*), Nile tilapia (*Oreochromis niloticus*), hogchoker (*trinectes maculatus*) [15], and hybrid striped bass (white bass *Morone chrysops* x striped bass *Morone saxatilis*) [7]. The recorded sound pressure levels (SEL_ss_, SEL_cum_, SPL_peak_) were comparable to the values measured in laboratory experiments using a high intensity controlled impedance fluid filled wave tube (HICI-FT) [6], [7], [15], [18] or a larvaebrator [5]. Accordingly, our sound measurements (SEL_ss_, SEL_cum_, SPL_peak_) confirm the sound pressure levels that have been used in the lab to mimic real time pile driving sound levels. Bearing in mind the expensive and challenging logistics to execute field experiments in addition to the difficulties to control all environmental parameters, laboratorial studies are a good approach.

### 2 Sound pressure thresholds

Exposure to sound pressures can lead to internal injuries. Sound pressure thresholds for the onset of injury in juvenile Chinook salmon (*Oncorhynchus tshawytsch*) were recommended as: SEL_cum_ = 210 dB re 1 µPa^2^.s derived from 960 strikes, each strike having a SEL_ss_ of 180 dB re 1 µPa^2^.s [6]. These values have been exceeded during pile driving of each monopile in our experiments. In an experiment using a HICI-FT, it was shown that after exposure to pile driving sounds, more individuals of a physoclist fish species were injured and the injuries per fish were more severe compared to a physostomous fish and a flatfish species, the latter lacking a swim bladder [6], [15]. These observations were strengthened by another experiment in the HICI-FT with the physoclist fish, the hybrid striped bass (white bass *Morone chrysops* x striped bass *Morone saxatilis*) [7]. Physoclist species are slower to change the volume of their swim bladder, which increases their vulnerability to high sound pressure levels [24]. Furthermore, more severe injuries and a higher number of injuries were reported in the larger size group of hybrid striped bass (white bass *Morone chrysops* x striped bass *Morone saxatilis*) physoclist fish (mean size 17.2 g) compared to smaller size group (mean size 1.3 g), [7]. This is in contrast to the current hypothesis that fishes less than 2 g are more susceptible to injury than larger fish when exposed to impulsive pile driving [7]. Internal sublethal injuries could also be present in the physoclist sea bass we used in our experiments, but this was beyond the scope of the present study.

Another experiment with the HICI-FT showed that fishes can heal from injuries as post-exposure time increased, after they were exposed to 960 strikes of 187 dB re 1 µPa^2^.s (SEL_cum_ = 217 dB re 1 µPa^2^.s) [7], [18]. However, internal injuries can also lead to mortality [6]. Mortal injuries in physoclistous hybrid striped bass of 1.3 g appeared at energy exposures with SEL_ss_ of 180 dB re 1 µPa^2^.s and SEL_cum_ of 210 dB re 1 µPa^2^.s for 960 strikes [7]. Similar levels did not result in increased mortality in our experiments. The mortality we noted, in any of the experiments, is more likely to be attributed to handling stress than to internal injuries. The sound pressure threshold causing mortality in juvenile sea bass less than 2 g lies above SPL_peak_ of 211 dB re 1 µPa, SEL_cum_ of 222 dB re 1 µPa^2^.s derived from 2312 strikes, and SEL_ss_ of 188 dB re 1 µPa^2^.s.

### 3 Acoustic particle motion

As shown above, the presence of sublethal or lethal injuries is correlated with the presence and type of a swim bladder, and sound ‘pressure’ seems to be the main sound component that induces the injuries. Nevertheless, the other underwater sound component ‘acoustic particle motion’ may also have an impact on the fishes, e.g. hearing damage, behavioural and masking studies [2], [25]. Although it is unlikely that particle motion has a direct effect on mortality, which is the topic of this paper, it is also influenced by pile-driving and literature is very scarce on particle displacement, velocity and acceleration in the ocean. Particle motion is a highly directional quantity and fishes may be able to determine the sound source direction [26]. While only those fishes that have evolved towards an acoustic coupling between their swim bladder (or other gas-filled structures) and their ear, can sense sound pressure, all fishes are able to sense particle motion [27], [28].

Shallow water acoustics are characterized by propagation complexities, like direct transmission, reflection and re-radiation [29], [30]. Therefore, potential effects of changes in the acoustic particle motion will only be seen in the near field of a sound source. Hence, it is recommended to measure both sound components in assessing all the effects of underwater sound.

### 4 Underwater sound in a wider perspective

The Marine Strategy Framework Directive (MSFD, Directive 2008/56/EC) aims at a good environmental status (GES) by 2020 for all European waters. One of the eleven descriptors concerns the impact of anthropogenic underwater noise. At present, there is insufficient knowledge on the impact of impulsive sounds to establish proper sound level thresholds for the marine environment. The US Fisheries Hydro-acoustic Working Group (FHWG) formulated interim criteria for maximum sound levels fishes could be exposed to without causing non-auditory tissue damage. The maximum SEL_cum_ for fishes weighing <2 gram was set at 183 dB re 1 µPa^2^.s and for fishes>2 gram at 187 dB re 1 µPa^2^.s [30]. Also, SEl_ss_ and the number of strikes should be taken into account, and the importance of particle motion should be explored further to develop sound criteria.

The sound pressure levels that were measured in this field study only represent a snapshot of the acoustic near field, with acoustic characteristics measured at a depth of 2.5 m below the sea surface and 45 m away from the sound source. Moreover, these *in situ* experiments can be seen as a worst case scenario in terms of exposure time (number of strikes) and sound levels. In the real world, young sea bass will be drifting with the currents through the wind farm construction zones, which influences the residence time in these zones. Most likely, the encounter time to very high sound levels (pressure and particle motion) will be too short to induce any physiological effects, except during slack tide when the water currents decrease to a minimum.

This *in situ* field study confirms and validates the mortality results found by laboratory experiments and contributes to our general understanding of the effects of pile driving sounds, a first step in the assessment process to establish sound criteria.

## Supporting Information

Data Figure S1 **SEL_ss_ in 1/3 octave band of three consecutive piling strike signals.** This file serves as an example of the SEL_ss_ spectrum in 1/3 octave bands (averaged over 0.7 s intervals) for three consecutive piling strike signals.
(PDF)

Data Figure S2 **Mean SEL_ss_ and SPL spectrum in 1/3 octave bands in presence and absence of pile driving.** This file contains the averaged SEL_ss_ values per 1/3 octave bands of the four sampled monopiles and the SPL values per 1/3 octave bands in the absence of pile driving.
(PDF)

Data Figure S3 **Monitoring of the immediate and delayed mortality of the exposed and control groups.** This file contains the daily mortality observed in the exposed and control groups up to 14 days after the experiment. In addition, mortality is expressed in mortality per day and cumulative mortality.
(PDF)
