# Supplementary material for: In Situ Mortality Experiments with Juvenile Sea Bass (Dicentrarchus labrax) in Relation to Impulsive Sound Levels Caused by Pile Driving of Windmill Foundations
Source: PLoS One. 2014 Oct 2;9(10):e109280. doi: 10.1371/journal.pone.0109280 (PMC4183662; doi:10.1371/journal.pone.0109280)
Supplement: Data Figure S1 — SELss in 1/3 octave band of three consecutive piling strike signals. This file serves as an example of the SELss spectrum in 1/3 octave bands (averaged over 0.7 s intervals) for three consecutive piling strike signals. (PDF) [file pone.0109280.s001.pdf]

**Event**	3005	3006	3007		
**Time**	1,03	2,55	4,05	**mean SELss**	**SELcum, practical**
**SELss**	183,50	181,47	182,78	182,584	217,4511378
**SPLpeak**	209,46	208,25	209,33		
**frequency peak**	125	125	125		
**1/3 octave band (Hz)**					
25	142,75	141,10	142,50		
31,5	145,49	144,00	145,28		
40	152,41	150,06	151,36		
50	152,85	150,73	152,27		
63	159,92	157,13	159,41		
80	164,07	161,84	163,94		
100	169,45	166,30	168,93		
125	176,44	173,55	176,05		
160	175,17	172,54	174,54		
200	167,52	166,41	168,10		
250	171,30	169,17	170,08		
315	168,17	166,58	167,57		
400	171,04	168,47	169,05		
500	173,42	169,16	171,31		
630	171,33	169,09	169,88		
800	167,30	168,06	168,61		
1000	168,15	166,43	166,04		
1250	169,67	166,57	168,05		
1600	168,75	165,29	166,67		
2000	167,80	167,68	166,71		
2500	166,10	168,06	169,12		
3150	165,10	168,29	168,56		
4000	165,38	166,35	165,37		
5000	164,52	163,69	162,40		
6300	164,20	163,81	162,64		
8000	164,28	162,84	161,87		
10000	162,12	160,73	160,03		
12500	160,25	159,24	158,88		
