# Supplementary material for: In Situ Mortality Experiments with Juvenile Sea Bass (Dicentrarchus labrax) in Relation to Impulsive Sound Levels Caused by Pile Driving of Windmill Foundations
Source: PLoS One. 2014 Oct 2;9(10):e109280. doi: 10.1371/journal.pone.0109280 (PMC4183662; doi:10.1371/journal.pone.0109280)
Supplement: Data Figure S2 — Mean SELss and SPL spectrum in 1/3 octave bands in presence and absence of pile driving. This file contains the averaged SELss values per 1/3 octave bands of the four sampled monopiles and the SPL values per 1/3 octave bands in the absence of pile driving. (PDF) [file pone.0109280.s002.pdf]

Figure 4: measured frequency spectra in the presence and absence of pile driving. Mean SELss of the total recorded piling strikes versus 1/3 octave bands and SPL of the control groups versus 1/3 octave bands.

	SELss (dB re 1 µPa².s)			
**1/3 octave bands (Hz)**	**C8**	**B3**	**G7**	**G8**
25	140,29	152,90	141,12	142,86
31,5	149,45	155,19	146,57	148,59
40	154,40	158,05	151,45	154,18
50	158,74	160,26	153,69	156,44
63	159,34	163,40	159,03	162,02
80	166,83	171,42	163,40	165,76
100	168,53	175,47	169,48	171,72
125	173,82	180,32	173,42	173,22
160	173,56	182,30	171,55	173,06
200	175,58	181,35	172,17	174,06
250	172,71	174,79	171,13	172,48
315	169,19	172,74	168,37	170,09
400	168,09	169,41	166,22	169,35
500	169,94	167,69	167,98	168,88
630	165,74	165,84	165,31	167,21
800	166,46	166,82	165,99	165,83
1000	164,03	164,62	163,53	164,71
1250	164,85	164,38	163,79	164,55
1600	163,94	163,75	163,84	164,11
2000	162,58	161,95	162,57	163,38
2500	161,59	160,93	161,33	162,39
3150	159,98	159,29	159,64	161,05
4000	158,46	158,58	158,16	159,81
5000	158,08	157,90	157,04	159,09
6300	157,41	157,25	156,12	158,09
8000	155,78	156,01	155,60	157,17
10000	153,77	154,09	154,30	155,63
12500	152,67	152,14	152,67	153,86

	SPL (dB re 1 μPa)			
**1/3 octave bands (Hz)**	**C1**	**C2**	**C3**	**C4**
25	131,62	117,51	134,40	126,19
31,5	131,83	115,53	137,98	125,61
40	128,35	113,56	142,49	125,02
50	123,74	113,42	136,03	124,70
63	122,10	112,67	128,61	123,83
80	121,52	111,25	123,42	123,55
100	122,52	111,40	116,94	128,43
125	122,72	113,18	114,72	127,12
160	122,08	110,50	113,17	122,91
200	120,98	110,36	114,26	122,21
250	121,55	112,60	114,70	119,39
315	121,21	115,16	119,73	117,93
400	119,62	112,64	112,38	117,83
500	117,75	110,82	110,08	116,20
630	117,20	112,05	112,38	113,77
800	116,00	109,99	108,57	112,70
1000	115,34	109,34	108,64	112,34
1250	115,42	110,61	109,59	112,72
1600	115,01	110,34	110,77	110,40
2000	115,19	111,93	112,03	111,11
2500	113,63	113,14	111,64	111,15
3150	113,53	113,09	108,52	110,99
4000	114,32	112,47	109,38	112,17
5000	111,97	112,71	112,49	109,01
6300	114,24	115,51	115,02	111,69
8000	114,64	116,65	115,79	112,27
10000	110,74	112,50	110,78	107,00
12500	110,55	115,76	112,89	104,93
