# Supplementary material for: In Situ Mortality Experiments with Juvenile Sea Bass (Dicentrarchus labrax) in Relation to Impulsive Sound Levels Caused by Pile Driving of Windmill Foundations
Source: PLoS One. 2014 Oct 2;9(10):e109280. doi: 10.1371/journal.pone.0109280 (PMC4183662; doi:10.1371/journal.pone.0109280)
Supplement: Data Figure S3 — Monitoring of the immediate and delayed mortality of the exposed and control groups. This file contains the daily mortality observed in the exposed and control groups up to 14 days after the experiment. In addition, mortality is expressed in mortality per day and cumulative mortality. (PDF) [file pone.0109280.s003.pdf]

Monitoring of the acute and delayed mortality of the exposed and control sea bass fish

* During the monitoring period of 14 days, extra samples of 12 fish were taken out of each aquarium on day 2, 5, 9
* This was done for weight and length measurements, they were stored on 7% buffered formaldehyde
* Fish of the second trip were not sampled during the monitoring period

		day 0				day 1	day 2	day 2	day 3	day 4	day 5		day 6	day 7	day 8
		fish in experiment	living fish immediately after exposure	fish sampled for further analyses just after experiment	living fish after sampling	living fish	**sampling**	living fish	living fish	living fish	living fish	**sampling**	living fish	living fish	living fish
**control**	**C1**	120	**0**	48	72	65	**12**	53	53	53	53	**12**	41	41	41
	**C2**	120	**0**	48	72	67	**12**	55	55	55	53	**12**	41	41	41
	**C3**	12	0	8	4	4		4	4	4	4		4	4	4
	**C4**	12	0	8	4	4		4	4	4	4		4	4	4
**exposed**	**C8**	120	**2**	48	70	67	**12**	55	55	55	55	**12**	43	43	43
	**B3**	120	**6**	48	66	65	**12**	53	53	53	50	**12**	38	38	38
	**G7**	12	0	8	4	4		4	4	4	4		4	4	4
	**G8**	12	0	8	4	4		4	4	4	3		3	3	3
	**% mortality**														
**control**	**C1**		0			9,72	0		0	0	0		0	0	0
	**C2**		0			6,94	0		0	0	3,64		0	0	0
	**C3**		0			0,00	0	0	0	0	0		0	0	0
	**C4**		0			0,00	0	0	0	0	0		0	0	0
**exposed**	**C8**		1,67			4,29	0		0	0	0		0	0	0
	**B3**		5			1,52	0		0	0	5,66		0	0	0
	**G7**		0			0	0	0	0	0	0		0	0	0
	**G8**		0			0	0	0	0	0	25		0	0	0
	**Cumulative mortality**														
**control**	**C1**		0			9,72	9,72		9,72	9,72	9,72		9,72	9,72	9,72
	**C2**		0			6,94	6,94		6,94	6,94	10,58		10,58	10,58	10,58
	**C3**		0			0	0	0	0	0	0		0	0	0
	**C4**		0			0	0	0	0	0	0		0	0	0
**exposed**	**C8**		1,6666667			5,95	5,95		5,95	5,95	5,95		5,95	5,95	5,95
	**B3**		5			6,52	6,52		6,52	6,52	12,18		12,18	12,18	12,18
	**G7**		0			0	0	0	0	0	0		0	0	0
	**G8**		0			0	0	0	0	0	25		25	25	25

		day 9	day 9	day 10	day 11	day 12	day 13	day 14	
		living fish	**sampling**	living fish	living fish	living fish	living fish	living fish	
**control**	**C1**	41	**12**	29	29	29	29	29	
	**C2**	41	**12**	29	29	29	29	29	
	**C3**	4		4	4	4	4	4	
	**C4**	4		4	4	4	3	3	jumped out of the aquarium
**exposed**	**C8**	43	**12**	31	31	31	31	31	
	**B3**	38	**12**	26	26	26	26	26	
	**G7**	4		4	4	4	4	4	
	**G8**	3		3	3	3	3	3	
	**% mortality**								
**control**	**C1**	0		0	0	0	0	0	
	**C2**	0		0	0	0	0	0	
	**C3**	0		0	0	0	0	0	
	**C4**	0		0	0	0	0	0	
**exposed**	**C8**	0		0	0	0	0	0	
	**B3**	0		0	0	0	0	0	
	**G7**	0		0	0	0	0	0	
	**G8**	0		0	0	0	0	0	
	**Cumulative mortality**								
**control**	**C1**	9,72		9,72	9,72	9,72	9,72	9,72	
	**C2**	10,58		10,58	10,58	10,58	10,58	10,58	
	**C3**	0		0	0	0	0	0	
	**C4**	0		0	0	0	0	0	
**exposed**	**C8**	5,95		5,95	5,95	5,95	5,95	5,95	
	**B3**	12,18		12,18	12,18	12,18	12,18	12,18	
	**G7**	0		0	0	0	0	0	
	**G8**	25		25	25	25	25	25	
